# The Employment of Consumptives

**Published:** 1917-02-24

**Authors:** 


### THE EMPLOYMENT OF CONSUMPTIVES.

LATELY an informal conference between representatives of several Government departments and public bodies has been held to consider a proposal by the medical adviser of the London County Insurance Committee for a national scheme for the after-care and employment of tuberculous persons. There is no doubt at all that such action would be beneficial, for up to the present the activities of local after-care committees and of the few farm colonies for consumptives that exist have been quite inadequate. Given suitable life conditions, the arrest of tuberculous disease in an individual, when once secured, can mostly be prolonged indefinitely. But what at this time of war strikes the reader first is that any steps taken, or to be taken, must be in what politicians call the measure of the possible. With money pouring out of the national coffers like water, and being absorbed as fast as it is effused by war needs, the resources available for non-military medical purposes cannot fail for a long time yet to be very limited. So much force is there in this consideration that we almost question the wisdom of holding deliberation just now, especially as the actual conditions after the war cannot be certainly predicted. There is nothing so demoralising, so chilling, so cynicism breeding, as dead-letter proposals and enactments, and of these sanitary medicine already has no few examples.

But, this proviso made, the plan itself is of course wholly commendable. One critic has expressed the hope that (to summarise his contention) there will be more centralisation in future and less devolution. Professor Osler has already made the same suggestion with reference to the anti-tuberculosis campaign. Besides the obvious evils of overlapping, there is the danger that local bodies will be found unfitted, in respect of special knowledge and other matters, to carry out the task well. Preventive medicine advances perceptibly every year. But the public benefits it offers cannot be secured by merely clapping on fifty or a hundred pounds a year to the salaries of medical officers of health, or to the already considerable ones of council or borough clerks, and entrusting them with all appertaining technical and administrative matters. If in the broad scheme more centralisation is necessary, in the details what is wanted is more devolution, more responsibility for the actual executants. The work of a medical officer of health is in one respect rapidly approaching that of a general medical practitioner, in being beyond the scope of any one man; for it ranges from water confervæ to midwifery, from plumbing to law, from fever hospitals to dentistry, ophthalmology and meteorology.

The present happens to be a good time for the recently recovered consumptive of the working class. He is exempt from military service, and has before him a labour market in which the demand is great and the supply ever growing less. For the first time then he can, to some extent, pick and choose suitable light outdoor employment. But this state of things will not last. It has been suggested aforetime in these columns—and the present food shortage strengthens the force of the advice—that the first step in initiating after-care of consumptives is to take advantage of the needs of the sanatorium whence they are discharged. Every sanatorium needs coal leading to it, and a supply of vegetables, bacon, fruit and eggs, and a certain amount of upkeep in the way of painting and light repairs. All this could be done by developing the grounds and employing paid ex-patients housed in huts on the premises. While thus healthfully and educatively occupied for a few months, these individuals have leisure to look out for suitable permanent employments. Under a national scheme all such, down to the smallest, could be largely reserved for the tuberculous.

